# Factors associated with patients’ healthcare-seeking behavior and related clinical outcomes under China’s hierarchical healthcare delivery system

**DOI:** 10.3389/fpubh.2024.1326272

**Published:** 2024-04-12

**Authors:** Lizhu Guo, Xin Du, Huanqi Wu, Shijun Xia, Jing Du, Xiangrong Kong, Xiaohui Yang, Chi Wang, Jianzeng Dong, Changsheng Ma, Lilly Engineer

**Affiliations:** ^1^Department of Arrhythmia Center, Beijing Anzhen Hospital, Capital Medical University, Beijing, China; ^2^Department of Health Policy and Management, Johns Hopkins Bloomberg School of Public Health, Baltimore, MD, United States; ^3^Data Science Academy, Capital University of Economics and Business, Beijing, China; ^4^Beijing Centre for Disease Prevention and Control, Beijing, China; ^5^Wilmer Eye Institute, School of Medicine, Johns Hopkins University, Baltimore, MD, United States; ^6^Heart Health Research Centre, Beijing, China; ^7^Department of Anesthesiology and Critical Care Medicine, Johns Hopkins University School of Medicine, Baltimore, MD, United States

**Keywords:** patients’ healthcare-seeking behavior, outcomes, hierarchical healthcare delivery system, cardiovascular risk factors, primary healthcare

## Abstract

**Introduction:**

The hierarchical healthcare delivery system is an important measure to improve the allocation of medical resources and promote equitable distribution of basic medical and health services. It is one of the key factors in the success or failure of China’s medical reform. This study aims to analyze the factors influencing patients’ healthcare-seeking behaviors, including socioeconomic and clinical outcomes, under China’s hierarchical healthcare delivery system, and to provide potential solutions.

**Methods:**

Patients receiving outpatient treatment in the past 14 days and inpatient care in the past 1 year were investigated. The multivariate logistic regression was used to analyze the influencing factors of patient’s medical treatment behavior selection, and to compare whether the clinical outcomes of primary medical institutions and grade A hospitals are the same.

**Results:**

Nine thousand and ninety-eight person-times were included in the study. Of these, 4,538 patients were outpatients, 68.27% of patients were treated in primary medical institutions; 4,560 patients were hospitalized, 58.53% chose to be hospitalized in grade A hospitals. Provinces and cities, urban and rural areas, occupation, education level, medical insurance type, income, whether there are comorbid diseases, and doctors’ medical behavior are the factors affecting the choice of medical treatment behavior. Patients who choose primary medical institutions and grade A hospitals have different control levels and control rate for the blood pressure, blood lipids, blood glucose.

**Conclusion:**

Under the hierarchical diagnosis and treatment system, the patients’ choice of hospital is mainly affected by their level of education, medical insurance types, and the inpatients are also affected by whether there are comorbid conditions. Clinical outcomes of choosing different levels of hospitals were different.

## Introduction

The hierarchical healthcare delivery system is one of the effective measures for countries to improve the allocation of medical resources and improve the efficiency of diagnosis and treatment (1, 2). In 2009, the CPC Central Committee and the State Council issued the Opinions on Deepening the Reform of the Medical and Health Care System, which proposed the concept of hierarchical diagnosis and treatment for the first time (3). In 2015, the General Office of the State Council issued the Guiding Opinions on Promoting the Construction of a Graded Diagnosis and Treatment System, actively exploring the establishment of a hierarchical diagnosis and treatment system across the country (4). Since China does not force patients to diagnose and treat according to the level, the proportion of medical treatment at the primary healthcare is still not ideal (5). Data shows that the current treatment rate of primary hospitals varies from 40–80% (6, 7). It is difficult to be treated in grade-A hospitals (8), and many of them are chronic patients (9), while the primary hospitals have very few patients there. Therefore, it is important to understand the influencing factors of patients’ choice of medical treatment behavior (10). Several studies have shown that (11–14) the main reason affecting patients’ choice of medical institutions is the quality of medical care. Past studies have assumed that the quality of chronic diseases treatment is the same in different medical institutions, but this may not be the case. Therefore, comparing the clinical indicators in different levels of medical institutions may find the main reasons affecting patient selection. This can guide the government departments to formulate corresponding policies and measures according to the actual influencing factors, which will help to solve the problem of low treatment rate of primary medical institutions in the hierarchical diagnosis and treatment.

## Materials and methods

The study adopted a two-stage stratified clustering design to ensure a representative and unbiased sample across various provinces, which is elaborated by the random selection of communities or villages within each selected province. Initially, one province is randomly selected from 7 geographical regions in China (Northeast, North, northwest, east, Central, South, and southwest). Beijing, Xinjiang Uygur Autonomous Region, Henan, Jilin, Guangdong, Yunnan, Jiangxi, and Zhejiang provinces were randomly selected for further sampling. All residents in selected communities (villages) were surveyed from June 1, 2014 to December 31, 2016. The patients with cardiovascular risk factors who are inpatient within 1 year and outpatient within 2 weeks were included in the study. A face-to-face structured interview were carried out by trained researchers using standard questionnaires and the physical examinations and laboratory tests were completed as well. In the morning, a fasting venous blood sample was taken for measurements of blood glucose, total cholesterol, low-density lipoprotein cholesterol (LDL-C) and triglycerides. The laboratory tests were carried out by Guangzhou Jinyu medical laboratory center Co., Ltd which was certified by the College of American Pathologists.

### Definitions

Cardiovascular risk factors and related diseases definitions: Factors (15) used in the 10-year CVD (CVD) risk model (China) included: age, mean systolic blood pressure, fasting total cholesterol, HDL cholesterol, current smoking (yes/no), diabetes (yes/no), body mass index (BMI), waist circumference, geographic area (northern/southern China), urban/rural residents and family history of CVD (16). Smoking status was defined as a self-reported non-smoker, former smoker (1 year), or current smoker. Diabetes was defined as fasting blood glucose 7.0 mmol/L, antidiabetic medication, or previous postprandial blood glucose 11.1 mmol/L, HbA1c 7%, or a diagnosis of diabetes. Weight, height, and waist circumference were measured by trained researchers using standard methods. Beijing, Xinjiang Uygur Autonomous Region, Henan and Jilin Province are defined as northern China, while Guangdong, Yunnan, Jiangxi, Zhejiang and other provinces are defined as southern China (17). Family history of CVD was defined as a history of coronary heart disease or stroke in any of the participant’s parents or immediate brother or sister. Hypertension was defined as measuring systolic blood pressure (SBP)140 mmHg, diastolic blood pressure (DBP) 90 mmHg (18), taking antihypertensive drugs, or self-reporting a previous diagnosis of hypertension. We defined SBP <130 mmHg and DBP< 80 mmHg as blood pressure control (19). Dyslipidemia was defined as total cholesterol 240 mg/dL, low-density lipoprotein cholesterol (LDL-C) 160 mg/dL, taking statins or other lipid-lowering drugs, or previously diagnosed as dyslipidemia. Cholesterol and blood glucose were measured in a central laboratory certified by the College of American Pathologists (Guangzhou Kimmer Testing Technology Co., Ltd).

### Questionnaire design

The questionnaire was designed by the China Clinical Research Center for Cardiovascular Disease (20) based on the national health service survey, the epidemic survey, the influential published questionnaires, and the opinions of several experts. For questionnaire validation, we conducted a presurvey of 400 respondents in a community, verified the internal and external validity of the questionnaire and modified it to the questions found. The final questionnaire includes: basic personal information, disease-related information, quality of life, health service utilization, clinical results and patient self-assessment, etc. (21).

### Theoretical framework

In our study, the analysis will be performed in the four dimensions of Andersen’s model (22) with further exploration of the interactions among the various dimensional factors.

The Anderson Healthcare Services behavior utilization model was established in 1968 by Ronald Max Anderson (23). It was originally used to analyze the influencing factors of home health service utilization. In the current Andersen’s model, after five modifications, patient medical care seeking behavior was influenced by four dimensions: situational characteristics, personal characteristics, medical behavior and outcome. There are interactions among these factors.

### Variable

The study data included situational characteristics (province and city, urban and rural), personal characteristics [age, gender, marital status, education level, family income and medical coverage, combination with hypertension, diabetes, dyslipidemia, coronary heart disease, stroke/transient ischemic attack (TIA) and atrial fibrillation; lifestyle factors included smoking and alcohol consumption]. Medical behavior (e. g., whether blood pressure is measured, whether to explain the significance of blood pressure values, and whether patients with chronic diseases prescribe corresponding drugs). And results (patient evaluation of their health status, current blood pressure (systolic and diastolic blood pressure), blood glucose, lipids, waist circumference, BMI levels), and disease awareness rate, treatment rate, and control rate.

### Quality control

Professor Du Xin, Professor Ma Changsheng and Professor Dong Jianzeng were responsible for designing the questionnaire and verifying the internal and external validity of the questionnaire. A group of regular personnel led by Guo Lizhu and Yang Xiaohui guide the national investigators to complete the questionnaire. Before the survey, the investigators were given unified training. For respondents with low education or illiterate, the investigator asked them questions and the questionnaire were filled out by the investigator. The study was approved by the ethics committee, and written informed consent was obtained from the respondents for each questionnaire. The questionnaire was answered on portable Android device (PAD), as a structured electronic questionnaire. The questionnaire is designed with reversed question verification the logical relationship, the numerical range is limited to avoid filling errors. If the questionnaire with contradictory answers, missed answers or errors cannot be submitted, and the errors are corrected on the spot. Effectiveness validation was performed after data collection by Du Jing, Wang Chi and Wu Huanqi. Xia Shijun made a preliminary analysis of part of the data.

### Statistical analysis

Through uni-variate analysis, compares the differences between patients in four dimensions: background characteristics, individual characteristics, health behaviors and outcome indicators, further incorporates statistically significant factors into the model, and explores the independent factors influencing selection of patients’ medical seeking behavior through multiple logistic regression analysis.

Categorical variables are shown as *n* (%), and continuous variables are shown as mean (SD). Continuous variables will be compared by using the unpaired t test, categorical variables by using the *χ*^2^ test. Using the SAS PROC SURVEYLOGISTIC procedure, with adjustment for sociodemographic and clinically relevant covariates, including age, sex, area of residence (urban versus rural), region, education level, household income, insurance status, and occupations, to assess the association between these factors and health seeking behaviors.

The clinical outcomes of blood pressure, blood glucose, and blood lipids in patients treated in primary healthcare and Grade-A hospitals were further compared.

## Results

The sampled community population is 47,841 people among which 24,344 people had risk factors for cardiovascular disease, 9,098 person-times had medical experience, 4,538 people visited outpatient clinics within 2 weeks, and 4,560 people had been hospitalized within 1 year. The statistics of urban and rural population according to outpatient visits and inpatient treatment and the number of people selected in this study by province are shown in Table 1.

**Table 1:** The number of participants in the national survey is grouped by urban/rural areas and provinces.

	*N*(%)	Primary healthcare		Grade-A hospital		Primary healthcare		Grade-A hospital	
Cities									
Rural	3,058	1,110	(35.84)	112	(7.77)	1,220	(64.52)	616	(23.08)
Urban	6,040	1987	(64.16)	1,329	(92.23)	671	(35.48)	2053	(76.92)
Provinces									
Beijing	1,185	444	(14.34)	421	(29.22)	48	(2.54)	272	(10.19)
Guangdong	1721	774	(24.99)	421	(29.22)	164	(8.67)	362	(13.56)
Henan	1,286	339	(10.95)	117	(8.12)	292	(15.44)	538	(20.16)
Jilin	1,088	221	(7.14)	70	(4.86)	352	(18.61)	445	(16.67)
Jiangxi	424	98	(3.16)	79	(5.48)	41	(2.17)	206	(7.72)
Xinjiang	805	168	(5.42)	28	(1.94)	375	(19.83)	234	(8.77)
Yunnan	1,556	427	(13.79)	117	(8.12)	552	(29.19)	460	(17.23)
Zhejiang	1,033	626	(20.21)	188	(13.05)	67	(3.54)	152	(5.7)

Table 2, by INPATIENTS choice of primary healthcare (PH) or grade A hospitals (GAH), the baseline characteristics, medical behaviors experienced, and some healthcare outcomes of the participants were listed. Among the 4,538 participants, 1,441 (31.75%) people chose the grade A hospital. Compared with patients who chose primary healthcare facilities, patients living in urban areas (PH: 43.79%; GAH: 29.29%), lives in northern China (PH: 37.85%; GAH: 44.14%), people engaged in non-manual labor (PH: 20.34%; GAH: 34.56%), high school education level or above (PH: 16.05%; GAH: 28.31%), the upper middle class of household income (50,000–70,000 yuan) (PH: 13.53%; GAH: 16.59%), with urban medical insurance (PH: 67.71%; GAH: 88.48%), combined with cardiovascular disease (PH: 3.07%; GAH: 6.04%), married person (PH: 83.76%; GAH: 85.01%), Han (PH: 96.22%; GAH: 97.29%) prefer to choose the grade A hospital. There were no significant differences in the gender or age of patients. In the course of medical behavior, patients who chose to visit primary healthcare received a higher proportion of blood pressure measurements during the visit (PH: 57.87%; GAH: 50.37%), the results of blood pressure measurement interpreted by the doctor (PH: 97.73%; GAH: 96.61%), but fewer correctly prescribed drugs were given (PH: 62.97%; GAH: 69.97%). A larger proportion of patients visited primary healthcare considered themselves to be healthy (PH: 94.30%; GAH: 92.62%), rate of blood pressure control is lower (PH: 64.13%; GAH: 71.20%).

**Table 2:** Characteristics of influencing factors (number and percentage) of INPATIENTS at cardiovascular risk factors when choosing different medical healthcare institution for treatment.

	Primary healthcare *n* = 3,097	Grade A hospital *n* = 1,441	*p*
**Environment**			
District (rural), %	1,110(35.84)	112(29.29)	<0.0001
Province (north), %	1,172(37.85)	636(44.14)	<0.0001
**Population characteristics**			
Gender (man), %	1,054(34.03)	468(32.48)	0.3015
Age (<65 years), %	1977(63.84)	922(63.98)	0.7952
Ethnic(han), %	2,980(96.22)	1,402(97.29)	0.2797
Marital status (married), %	2,594(83.79)	1,225(85.01)	0.0185
Occupation (mental), %	630(20.34)	498(34.56)	<0.0011
Education (below junior high school), %	2,156(66.66)	753(57.01)	<0.0001
Income (5–70,000), %	419(13.54)	239(16.61)	<0.0001
Health insurance (urban health insurance), %	2097(67.71)	1,275(88.48)	<0.0001
Disease (CHD), %	95(3.07)	87(6.04)	0.0002
**Health behavior**			
Measure BP	1,659(53.57)	682(47.33)	<0.0001
Inform the patient of HT	2,802(97.73)	1,308(96.60)	0.1878
Treated rate of HT, %	1,170(62.97)	557(69.97)	0.0005
**Outcome**			
Self evaluation (healthy), %	2,911(94.30)	1,331(92.62)	<0.0001
HT awareness rate	1,387(74.65)	608 (76.38)	0.1014
Hypertension control rate	747(40.20)	381(47.86)	0.0003

Table 3, by INPATIENTS ‘choice of primary healthcare or grade A hospitals, the baseline characteristics, medical behaviors experienced, and some healthcare outcomes of the participants were listed. Among the 4,560 participants, 2,669 (58.53%) people chose the grade A hospital. Compared with patients who chose primary healthcare facilities, patients living in urban areas (PH: 35.48%; GAH: 76.92%), lives in southern China (PH: 43.58%; GAH: 44.21%), male (PH: 41.85%; GAH: 36.28%), people engaged in non-manual labor (PH: 11.42%; GAH: 24.73%), high school education level or above (PH: 15.66%; GAH: 34.03%), the upper middle class of household income (3–70,000 yuan) (PH: 25.30%; GAH: 35.92%), with medical insurance and which is not the new rural cooperative medical insurance (PH: 38.66%; GAH: 72.35%), combined with coronary heart disease (CHD) (PH: 6.28%; GAH: 11.13%), Han (PH: 88.74%; GAH: 95.28%) prefer to choose the grade A hospital. There were no significant differences in the marital status or age of patients. In the course of medical behavior, INPATIENTS who chose to visit grade A hospitals received result interpretation of their blood pressure measurements less than primary healthcare during the hospitalization (PH: 97.92%; GAH: 97.16%), patient in grade A hospital more correctly prescribed drugs were given (PH: 49.55%; GAH: 62.40%). There is no difference in the proportion of patients given by doctors to measure blood pressure in primary healthcare and grade A hospitals. Patients attending grade A hospitals perceived their health to be worse (PH: 6.52%; GAH: 7.75%), more people know they have high blood pressure (PH: 75.07%; GAH: 70.89%), higher blood pressure control rate (P: 32.87%; GAH: 36.65%).

**Table 3:** Characteristics of influencing factors (number and percentage) of INPATIENTS at cardiovascular risk factors when choosing different medical healthcare institution for treatment.

	Primary healthcare *n* = 1891	Grade A hospital *n* = 2,669	*p*
**Environment**			
District (rural), %	1,220(64.52)	616(23.08)	<0.0001
Province (north), %	1,067(56.42)	1,489(55.79)	<0.0001
**Population characteristics**			
Gender (man), %	686(36.28)	1,117(41.85)	0.0001
Age (<65 years), %	1,061(56.11)	1,451(54.36)	0.1880
Ethnic(han), %	1,678(88.74)	2,543(95.28)	<0.0001
Marital status (married), %	1,566(82.86)	2,225(83.40)	0.1707
Occupation (mental), %	216(11.42)	660(24.73)	0.0526
Education (below junior high school), %	1,594(84.34)	1760(65.97)	<0.0001
Income (3–70,000), %	478(25.30)	958(35.92)	<0.0001
Health insurance (urban health insurance), %	731(38.66)	1931(72.35)	<0.0001
Disease (CHD), %	129(6.82)	297(11.13)	0.002
**Health behavior**			
Measure BP	726(40.31)	1,097(42.27)	0.2019
Inform the patient of HT	1,693(97.92)	2,423(97.16)	<0.0001
Treated rate of HT, %	606(49.55)	1,059(62.40)	<0.0001
**Outcome**			
Self-evaluation (healthy), %	1751(93.48)	2,449(92.25)	0.0121
HT awareness rate	867(70.89)	1,274(75.07)	0.0066
Hypertension control rate	402(32.87)	622(36.65)	0.0005

**Figure 1:**
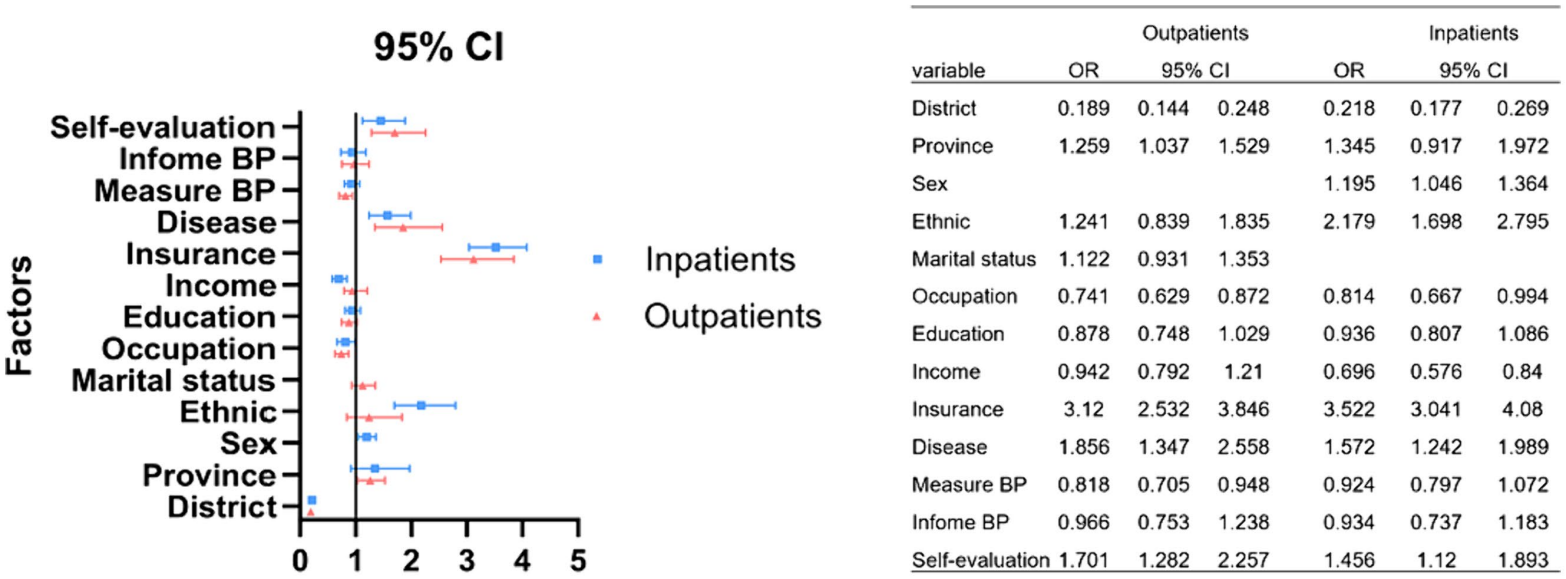
Analysis of the influencing factors for patients at risk of cardiovascular disease when choosing different medical institutions for treatments.

Figure 1 shows the results of binary logistic regression analysis of factors affecting patients’ medical behavior choices. Whether INPATIENT OR OUTPATIENT, urban and rural areas, their own health evaluation, combined diseases, their own occupation and type of medical insurance are the factors that influence patients’ choice of medical treatment. Their resident city and whether the doctor measure the patient’s blood pressure at the time of the visit are the factors that affect the patient’s OUTPATIENT visit. The amount of income, ethnic and gender affects the choice of INPATIENT hospitalization medical behavior.

**Figure 2:**
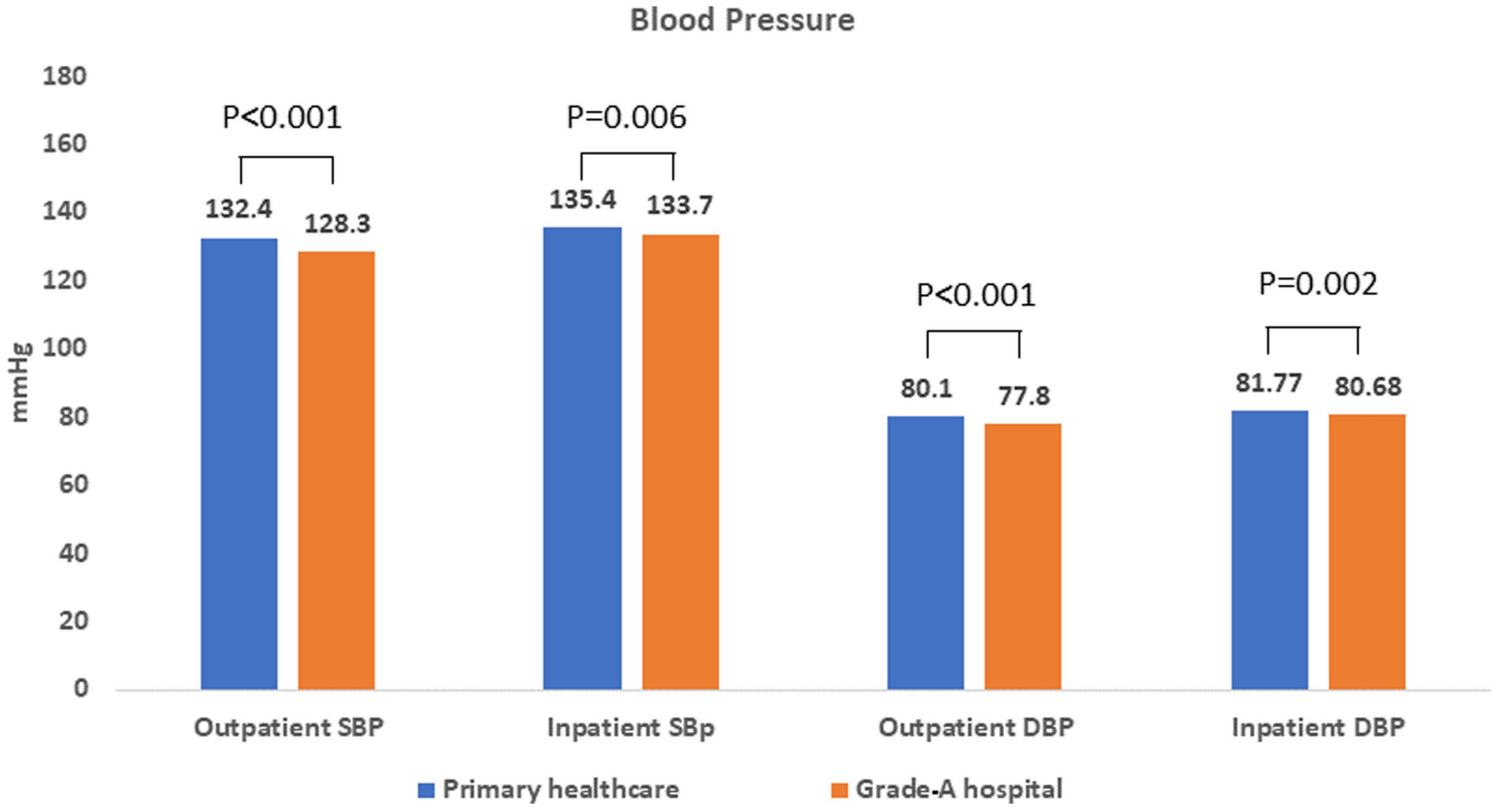
Blood pressure of patient visits to different levels of medical institutions.

Figure 2 shows the current blood pressure levels of patients who chose primary healthcare or grade A hospitals being OUTPATIENT and INPATIENT, respectively. It can be seen that patients at different levels of medical institutions, the patients’ blood pressure difference, whether systolic or diastolic, whether OUTPATIENT or INPATIENT. Patients have better blood pressure levels after treatment in grade A hospitals (SBP 128.3 ± 18.3 mmHg, DBP 77.8 ± 10.2 mmHg), among which OUTPATIENTS who visited grade A hospitals have the best blood pressure control, and the average value reaches the target blood pressure (≤130/80 mmHg) (M).

Cohen’s kappa was employed to ensure consistency in hypertension diagnosis among patients, yielding high agreement rates (0.9793 for outpatients and 1 for inpatients).

**Figure 3:**
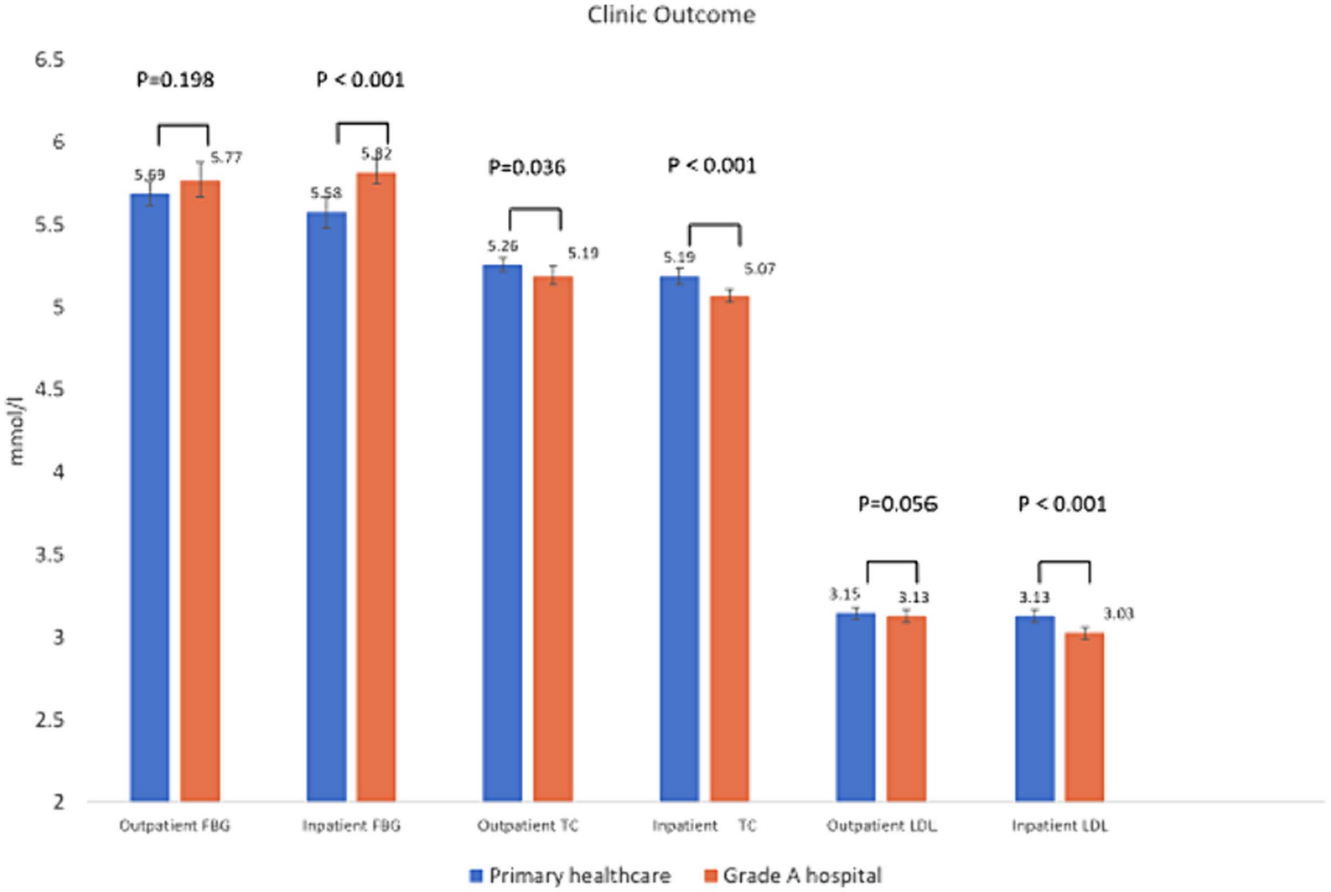
Blood glucose and lipid status of outpatient visits to different levels of medical institutions.

Figure 3 shows the current clinical outcomes of patients who chose primary healthcare and grade A hospitals being OUTPATIENT AND INPATIENT respectively: blood glucose, total cholesterol, LDL cholesterol. It can be seen that INPATIENTS at different levels of hospitals have different levels of blood glucose, total cholesterol, and LDL cholesterol levels. The patients have better clinic outcome after treatment in grade A hospitals (TC_OUTPATIENT_ 5.19 ± 1.00 mmol/l, TC_INPATIENT_ 5.07 ± 1.05 mmol/l; LDL_INPATIENT_ 3.03 ± 0.87 mmol/l) and lower levels of total cholesterol and LDL cholesterol after outpatient treatment in grade A hospitals (LDL_OUTPATIENT_ 3.13 ± 0.85 mmol/l). The former is statistically different. Whether OUTPATIENT AND INPATIENT, current blood glucose after treatment at different levels of medical healthcare is up to standard.

## Discussion

In this study, the rates of outpatient and inpatient visits primary hospitals with cardiovascular risk factors were 68.27 and 41.47% respectively, which were lower than the policy target proposed by the Chinese government in 2015 (70%). We found that the main factors influencing the choice of patient medical treatment behavior are occupation, education, reimbursement method, income, and whether the patient had a comorbid illness. Secondly, marital status, living habits and body shape affect the choice of OUTPATIENT medical treatment. Gender and ethnic group influence the INPATIENTS’ choice of hospitalization (24).

Hierarchical diagnosis and treatment refer to the classification according to the severity of diseases and the level of difficulty of treatment, the medical institutions of different levels undertake the treatment of different diseases (25–27). Mix all the diseases together to discuss the effect of hierarchical diagnosis and treatment is not effective nor conducive to identify the potential reasons affecting patients’ behavioral choice.

This study was sampled in the national wide communities and focused on patients with CVD risk factors, the vast majority of whom were with hypertension. This is conducive to the analysis of the medical behavior of cardiovascular patients who should go to the primary hospital for diagnosis and treatment. As is known to all, cardiovascular disease is the first burden disease in China which has the large number of patients and was the heavy medical burden (27–29). Patients with chronic cardiovascular diseases such as hypertension did not act in according to the recommended program (primary medical healthcare treatment) is one of the main reasons leading to the failure of graded diagnosis and treatment (30, 31).

Anderson’s theoretical framework (32) shows that patient medical treatment behavior choice is influenced by four dimensions: environmental factors, personal factors, medical behavior and medical outcome. For such significant number of hypertension patients, on the facts of the extensive countrywide publication, the diagnosis and treatment costs are not high, and almost all medical institutions can provide treatment, why there are still many patients choose the grade-A hospital? It is worth our thinking.

Hypertension is a common disease, a high incidence of disease, when we do the evaluation of health service utilization, we often assume that the clinical effect of treating hypertension in any medical institution to treat hypertension is the same, but is it really the case? (33, 34).

In addition to the questionnaire, in this study we also measured the clinical indicators of the patients, which enabled us to evaluate the control effect of the disease. Through the data, we found that the blood pressure and lipid levels of patients in primary medical institutions were higher than those of patients in grade A hospitals. The awareness rate, treatment rate and control rate of hypertension and hyperlipidemia were even lower in primary hospitals (35). The difference in clinical outcomes may be the underlying reason why patients choose a grade A hospital instead of a primary medical institution. As some studies have shown, medical treatment in China is the mode of individual free choice, and whether patients can be cured is the primary factor that patients consider when making medical choices (13).

The findings in this study will facilitate us to formulate policies and effective measures for the hierarchical diagnosis and treatment of hypertensive patients (36). Improving the effectiveness of hypertension diagnosis and treatment in primary medical institutions will be the goal, which can be achieved by increasing the training of medical staff, formulating standardized diagnosis and treatment procedures, digitizing the management of patients’ blood pressure data, and AI checking the accuracy of prescriptions.

This study has some limitations, such as: the study data are obtained from a structured questionnaire. In the future, semi-structured interviews will provide better insight into the factors and perceptions of patients’ health seeking behaviors. The reliability of the questionnaire can be further evaluated using the Cronbach’s alpha. And a standard quality of life assessment questionnaire will provide more information. To expand the variety of diseases in the attending patients to perform a multi-level aggregation analysis will provide more refined evidence for policy making.

## Conclusion

The choices of medical behavior of patients with cardiovascular disease risk factors are not only influenced by socioeconomic factors but also the differences in clinical outcomes of treatment which might be the underlying cause especially for chronic diseases such as hypertension.

## Data availability statement

The original contributions presented in the study are included in the article/supplementary material, further inquiries can be directed to the corresponding author/s.

## Ethics statement

The studies involving humans were approved by the Ethics Committee of Beijing Anzhen Hospital affiliated to Capital Medical University (IRB No. 2013024). The studies were conducted in accordance with the local legislation and institutional requirements. The participants provided their written informed consent to participate in this study.

## Author contributions

LG: Writing – original draft, Project administration. XD: Conceptualization, Writing – review & editing. HW: Data curation, Writing – review & editing. SX: Formal analysis, Writing – review & editing. JD: Data curation, Writing – review & editing. XK: Data curation, Writing – review & editing. XY: Project administration, Writing – review & editing. CW: Visualization, Writing – review & editing. JZD: Project administration, Writing – review & editing. CM: Resources, Writing – review & editing. LE: Writing – original draft, Writing – review & editing.
